# Risk assessment of cholera outbreaks in Eastern Visayas, Philippines, 2024

**DOI:** 10.5365/wpsar.2026.17.3.1271

**Published:** 2026-07-14

**Authors:** Soonjong Bae, Boyd Roderick Cerro, Angelito Umali, Lalaine Villasis, Kathleen Ryan, Bonifacio Magtibay, Albert John Enrico Dominguez, Harriette Linelle Cruz, Andrea Gail Palagbas, Rez Dacoco, Percival De Paz, Ludina L Insigne, Marvin Jed C Soledad, Jody Nesus, Nayco Yap, Eleanor Salvador-Peña, Katherine Gamiao, Ian Christian Gonzales, Yui Sekitani

**Affiliations:** a World Health Organization Representative Office for the Philippines, Manila, Philippines.; b Center for Health Development Eastern Visayas, Eastern Visayas, Philippines.; c United Nations Children's Fund, Manila, Philippines.; d Epidemiology Bureau, Department of Health, Manila, Philippines.

## Abstract

**Objective:**

This study presents the findings of a joint cholera risk assessment conducted in Eastern Visayas, the region in the Philippines with the highest incidence of the disease in 2022–2023. The study aimed to identify key risk factors, review progress since a 2016 assessment, and highlight persistent gaps in prevention, preparedness and response.

**Methods:**

In July 2024, the World Health Organization, the United Nations Children’s Fund and the Philippine Department of Health conducted a joint assessment using a standardized checklist for focus group discussions and interviews across four technical areas: partnership and coordination; epidemiology and surveillance; water, sanitation and hygiene (WASH); and risk communication and community engagement. Epidemiological trends between 2022 and 2024 were analysed, and water samples from wells and refilling stations were tested for *Escherichia coli* and total coliforms.

**Results:**

From 2022 to 2024, 12 284 cholera cases were reported in Eastern Visayas (case fatality rate: 0.5%). Testing showed 100% contamination in Level I and II sources, while 33% of Level III samples tested positive for total coliforms despite no *E. coli* detection. Partial progress has been made in implementing the 2016 assessment’s recommendations, with good practices in surveillance, water sampling and community awareness, although gaps remain in outbreak response, coordination and WASH access.

**Discussion:**

This assessment identified both progress and persistent challenges across the four technical areas. Key limitations were found in the qualitative design and the limited geographical coverage. Conclusions focused on prioritizing the strengthening of national cholera strategies by embedding them within the broader Food and Waterborne Diseases Prevention and Control Program alongside local ownership and equity-focused WASH investments.

Cholera is a highly transmissible disease spread through contaminated food or water, and can cause death within hours. (*1*, *2*) Cholera outbreaks continue to affect populations with limited access to sanitation, particularly in developing countries and regions affected by poverty, displacement, conflict and climate change. (*3*, *4*)

Despite global efforts to reduce the spread of disease, transmission persists, with 472 697 cases reported to the World Health Organization (WHO) in 2022 and 535 321 in 2023. (*5*) The true burden is expected to be much greater, with 1.3–4 million estimated cases and 21 000–143 000 deaths annually. (*6*)

In 2023, the Philippines reported 3982 cholera cases and 19 deaths to WHO. (*5*) The Eastern Visayas region recorded the highest incidence among all 17 regions in both 2022 and 2023 (Department of Health, Philippines, Epidemic-prone Disease Case Surveillance [EDCS] report, morbidity weeks 52, 2022, and 48, 2023, respectively. Unpublished data). The region has a history of significant outbreaks of cholera and acute watery diarrhoea (AWD); in June 2016, Samar Province declared an AWD outbreak, with 1573 cases and 32 deaths. (*7*) In response, fellows from the Department of Health (DOH) Field Epidemiology Training Program conducted epidemiological investigations, (*8*) while WHO and DOH conducted a joint risk assessment that resulted in the 2016 recommendations evaluated in this study (**Table 1**).

**Table 1:** Recommendations from the 2016 cholera risk assessment and 2024 assessment of their implementation, by domain

2016 recommendation	2024 implementation level	Progress notes
**Partnership and coordination**		
**Strengthen coordination and partnerships within the EOC for effective outbreak response.**	**Partially implemented**	**While the EOC has been established as a central command hub, further improvements are needed to clarify its operational role through the development of comprehensive TOR and SOPs.**
**Establish the EOC as a central command hub and create comprehensive TOR and SOPs to clarify its operational role.**		
**Establish regular Incident Command System meetings to reinforce the EOC's function as a multidisciplinary centre for consolidating and documenting information from various sources.**	**Partially implemented**	**While Incident Command System meetings have been conducted to reinforce the EOC’s function as a multidisciplinary centre, further improvements are needed to consolidate and document information from various sources.**
**Conduct regular multisectoral coordination meetings, involving the surveillance and epidemiology, WASH and RCCE clusters to ensure coordinated actions, information sharing, and the regular dissemination of situation reports to maintain robust partnerships and support collective response efforts.**	**Partially implemented**	**While multisectoral cluster meetings involving surveillance and epidemiology, WASH and RCCE are conducted as part of the cholera response, they are not systematically convened for all events.**
**Relocate the EOC to a flood-free area optimized for communication, with fully operational equipment.**	**Not implemented**	**Formal planning for relocation and equipment upgrading has not been initiated.**
**Epidemiology and surveillance**		
**Enhance case-based reporting by integrating a suspected case definition for AWD as a notifiable condition within the Philippine Integrated Diseases Surveillance and Response system.**	**Partially implemented**	**Although AWD is included in the case definition for suspected cholera, which is a notifiable disease, AWD itself has not yet been designated as a notifiable condition.**
**Reclassify cholera as a Category 1 notifiable condition, requiring reporting within 24 hours.**	**Not implemented**	**There have been no formal administrative discussions or policy reviews initiated regarding the reclassification of the disease status.**
**Implement comprehensive risk assessment and outbreak response training at all administrative levels, with an emphasis on timely, adaptable local risk assessments.**	**Not implemented**	**While general outbreak response trainings have been conducted, specific capacity building for timely and adaptable local risk assessments has not yet been implemented.**
**Enhance event-based surveillance at all levels and develop a risk matrix to prioritize high-risk villages for focused interventions.**	**Partially implemented**	**While event-based surveillance has been enhanced at all levels, the development of a risk matrix to prioritize high-risk villages for focused interventions has not yet been implemented.**
**WASH**		
**Improve supply systems to reduce contamination, promote household water treatment and safe storage, and establish regular water quality monitoring in high-risk areas.**	**Partially implemented**	**Training on water safety planning was initiated in 2016–2017 in collaboration with partners and remains an ongoing requirement for water districts.**
Advise the public to avoid water sources that test positive for ***Escherichia coli***, along with addressing water supply issues in health-care facilities.	**Partially implemented**	**WASH communication began in 2016, and the implementation of the WASH FIT plan in the region is underway. Additionally, municipal health offices in selected provinces have been trained in the application of WASH FIT with support from UNICEF.**
**Maintain clean drinking-water containers, expand access to toilets and handwashing facilities, and implement solid waste management in schools through collaboration with the Department of Education.**	**Partially implemented**	**The implementation of the Department of Education’s WinS Program Three-Star Approach in Eastern Visayas has been supported through capacity-building and technical assistance provided by UNICEF, in collaboration with the National WinS Technical Working Group. From school year 2017–2018 to 2023–2024, the proportion of public schools in the region with at least a one-star rating increased significantly, from 2% to 61% (representing schools achieving at least a one-star rating out of the total schools participating in the national WinS monitoring).^a^**
**Update and disseminate best practices from the Philippine Approach to Sustainable Sanitation program, launched by UNICEF post-Typhoon Haiyan.**	**Partially implemented**	**The Philippine Approach to Sustainable Sanitation training and programmatic support were provided to Samar and Northern Samar provinces, enabling them to achieve more than 12 municipalities and multiple villages with zero open defecation. Additionally, orientation on the Approach for other provinces in Eastern Visayas was conducted in collaboration with the National Economic and Development Authority – Eastern Visayas and the Regional Development Council.**
**Establish Local Drinking-water Quality Monitoring Committees and enhance data management systems for WASH activities.**	**Partially implemented**	**Several local government units have established their committees, with capacity-building efforts planned for implementation. In Eastern Visayas, local drinking-water quality monitoring committees have also been set up with support from UNICEF.**
**RCCE**		
**Reinforce messages on safe water practices and expand information access through media.**	**Fully implemented**	**WHO, in collaboration with DOH, produced a 30-second commercial on safe water the previous year.**
**Monitor and evaluate communication activities, enhancing DOH staff capacity in risk communication.**	**Partially implemented**	**RCCE trainings have been conducted, but only at the staff level; plans for senior-level training are currently underway. Staff-level personnel include health promotion officers, nurses and other implementers responsible for carrying out health promotion and RCCE initiatives at the local level. Senior-level officials, on the other hand, serve as decision-makers and institutional spokespersons, including regional directors and assistant regional directors.**
**Provide targeted training for managers and senior staff.**	**Partially implemented**	**The DOH Health Promotion Bureau, in collaboration with USAID and WHO, has conducted national and subnational capacity-building activities for RCCE in health emergencies. In 2023, the DOH and WHO jointly organized a training on crisis communication and infodemic management training. The workshop was attended by managerial and senior staff from various centres for health development, including regional directors and assistant regional directors. However, a training specifically dedicated to managers and senior officials has yet to be conducted.**
**Promote exclusive breastfeeding to prevent diarrhoea, and educate water vendors on maintaining water quality at refilling stations, with regular monitoring by local government units.**	**Not implemented**	**DOH has implemented breastfeeding campaigns; however, they are not explicitly linked to diarrhoeal disease prevention.**
**Carry out ongoing IEC campaigns across communities, schools and health-care facilities to promote basic hygiene and sanitation practices.**	**Fully implemented**	**The DOH Central Office successfully cascaded IEC materials to the subnational level. Regional offices were provided with budgets to produce these materials and disseminate them in various settings, including schools and communities. During the field visits, IEC materials were observed to be visibly displayed in these locations.**
**Teach the community how to prepare homemade oral rehydration solution.**	**Partially implemented**	**DOH has produced IEC materials, including videos, on how to prepare oral rehydration solution. However, based on interviews with some village health workers, there is a continued need for dedicated community education, as some community members still lack knowledge and skills on oral rehydration solution preparation.**

AWD: acute watery diarrhoea; DOH: Department of Health; EOC: emergency operations centre; IEC: information, education and communication; RCCE: risk communication and community engagement; SOPs: standard operating procedures; TOR: terms of reference; UNICEF: United Nations Children's Fund; USAID: United States Agency for International Development; WASH: water, sanitation and hygiene; WASH-FIT: Water and Sanitation for Health Facility Improvement Tool; WHO: World Health Organization; WinS: Water, Sanitation and Hygiene in Schools.

^a^ Department of Education, Philippines; 2024. (*9*)

*Source:* 2016 cholera risk assessment. Manila: World Health Organization/Department of Health, Philippines; 2016. Unpublished internal report.

In January 2024, the DOH Eastern Visayas Center for Health Development (EVCHD) requested a new cholera risk assessment from WHO following outbreaks in 2022 and 2023. A joint mission was conducted in July 2024 by WHO, the United Nations Children’s Fund (UNICEF), the DOH Epidemiology Bureau and EVCHD. Three primary risk questions guided this assessment: What factors sustain cholera transmission in Eastern Visayas, what progress has been made, and which gaps have remained across key technical areas since 2016?

## Methods

### Site selection

EVCHD identified cities and municipalities for the risk assessment based on whether any confirmed cholera case or cholera outbreak had been recorded since 1 January 2022.

### Risk assessment team

A total of 18 members participated in the mission, including six from WHO, four from UNICEF and eight from EVCHD. They were divided into two teams, each comprising technical focal points for surveillance and epidemiology; water, sanitation and hygiene (WASH); and risk communication and community engagement (RCCE). Teams were supported by local focal persons from the assessment sites.

### Risk assessment procedures

The assessment was conducted from 8 July (day 0) to 12 July 2024 (day 4). On day 0, teams convened with EVCHD leadership to finalize the methodology. The assessment applied an operational multisectoral approach.

**Fig. 1:**
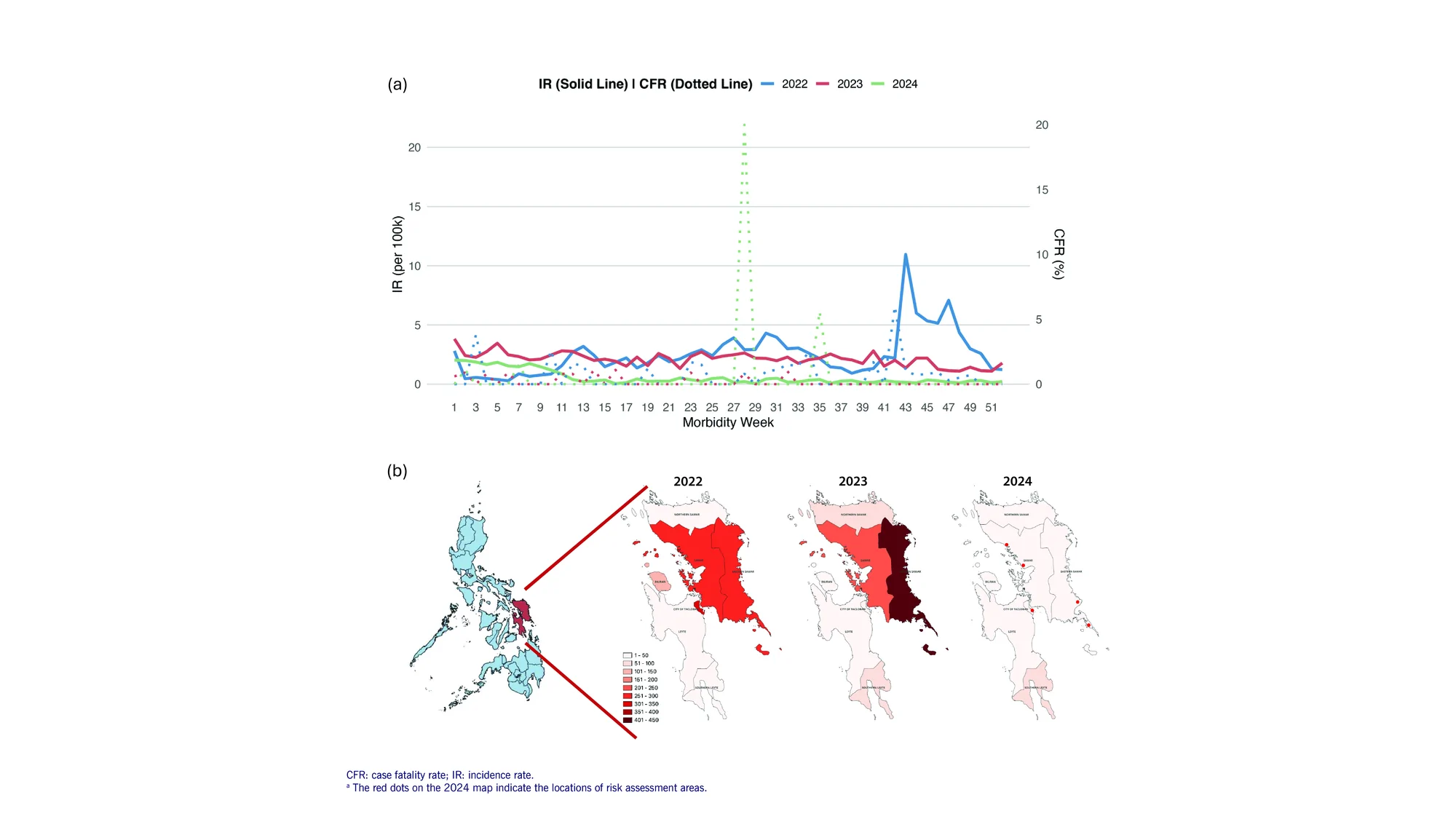
Trends in cholera incidence/100 000 population and CFR by: (a) morbidity week; and (b) spatial distribution of incidence,a Eastern Visayas, Philippines, 2022–2024

On 9–10 July, assessments were conducted in separate geographical areas, with team 1 in Eastern Samar and team 2 in Samar, followed by a joint assessment in the City of Tacloban in Leyte on 11 July (**Fig. 1**).

[**insert** Figure 1]

A standardized risk assessment checklist was developed by the teams and systematically applied across all technical areas (**Supplementary Materials 1–3**). Qualitative data were collected through focus group discussions and interviews that sought representation from all relevant technical categories, including municipal health officers, village health workers, sanitary inspectors, disease surveillance officers and other health providers, as well as community members. Data were collected in English, Filipino, and the local dialects Visayan and Waray. These procedures applied to all technical areas, including RCCE. Information about partnerships and coordination was captured through cross-cutting items embedded within the checklists.



### Water sampling and testing

To assess water source contamination at the assessment sites, environmental sampling was conducted. Using purposive sampling, a total of 19 water samples were collected from Level I, II and III water sites reported to have high cholera incidence. In the Philippines, Level I refers to sources such as protected wells or developed springs without distribution systems; Level II consists of communal faucet systems connected to a piped distribution network; and Level III is a waterworks system that provides piped connections directly to households. (*10*) All samples were analysed using Colilert-18 (IDEXX, Westbrook, ME, USA) enzyme-substrate water testing kits to determine the presence of total coliform and *Escherichia coli*, following the Philippine National Standards for Drinking-water of 2017 (*11*) and standard methods for water and wastewater testing. (*12*)

Samples were collected from drinking-water sources, storage facilities and distribution lines following DOH standard procedures. They were processed within a maximum holding time of 6 hours after collection and then placed in portable incubators at 35 °C for 18 hours. The results were interpreted based on colour changes: colourless (negative), yellow (positive for total coliforms) and yellow with fluorescence under ultraviolet light (positive for *E. coli*) using Colilert-18 and ColiKat (ColiKat, Seoul, Republic of Korea) systems. (*13*)

### Epidemiological data and case definitions

A descriptive analysis was conducted to assess epidemiological trends from 2022 to 2024 using an EVCHD Regional Epidemiology and Surveillance Unit (RESU) line list, which included all suspected, probable and confirmed cholera cases and deaths.

Case definitions followed national surveillance frameworks, and these changed over time. Between 2008 and 2021, a suspected case was a person aged ≥ 5 years presenting with AWD, and a confirmed case required laboratory confirmation of *Vibrio cholerae* O1 or O139. (*14*)

In 2022, definitions transitioned to the EDCS guidelines, which required that a case be aged ≥ 2 years and have signs of severe dehydration in addition to AWD. Then, during the large outbreaks from February 2022 to March 2024, a broader definition was temporarily adopted to support the response: a suspected case was defined as anyone aged ≥ 2 years with AWD but without the requirement for severe dehydration; a probable case was a suspected case with a positive result from a rapid diagnostic test (RDT); and a confirmed case required laboratory confirmation. (*15*) After case numbers declined in March 2024, the region reverted to the EDCS definition of cases in non-endemic areas, which required severe dehydration.

### Data analysis and visualization

To characterize the outbreak, weekly aggregated cases, deaths, spatial distribution maps and case fatality rates (CFRs) were calculated for each morbidity week. The annual incidence was estimated using population denominators derived from the 2023 Philippine Statistical Yearbook based on 2020 census projections. (*16*)

All identifiable data were anonymized in line with ethical standards. Spatial analysis was conducted in QGIS open-source software v. 3.40 (https://​qgis​.org), and all other analyses were done in RStudio 2024.04.2+764 (R Core Team, Vienna, Austria; https://​www​.r​-project​.org/​about​.html).

To assess implementation of the 2016 recommendations, findings from the 2024 assessment were systematically compared with each recommendation and grouped into four technical areas: partnership and coordination, epidemiology and surveillance, WASH and RCCE. Implementation status was categorized as “not implemented,” “partially implemented” or “fully implemented,” with accompanying narrative notes (**Table 1**).

### Synthesis of findings and reporting

On 11 July 2024, technical teams consolidated their findings and formulated recommendations. On 12 July, key summary findings and recommendations were presented to the EVCHD regional director and other stakeholders.

## Results

### Cholera epidemiology

Between 2022 and 2024, Eastern Visayas reported a total of 12 284 cholera cases, with 5959 cases in 2022, 5029 in 2023 and 1296 in 2024. Altogether, 60 deaths occurred over this period (44 in 2022, 11 in 2023, 5 in 2024), yielding an overall CFR of 0.5%. Samar recorded the highest cumulative burden over the 3 years (4457 cases; 33 deaths; CFR: 0.7%), followed by Eastern Samar (3454 cases; 4 deaths; CFR: 0.1%) and Leyte (1513 cases; 12 deaths; CFR: 0.8%).

The annual cholera incidence for 2022–2024 showed Eastern Samar in 2023 with the highest incidence (410/100 000 population), followed by the City of Tacloban in 2022 (345) and Samar in 2022 (296) (**Fig. 1**).

### Review of 2016 recommendations

Of the 20 recommendations from the 2016 assessment, only two were fully implemented, both of which were in the RCCE category. While most other recommendations across all categories were partially implemented, including all five WASH-related actions, key areas in partnership and epidemiology still face implementation gaps (**Table 1**).

### Partnership and coordination

The 2024 risk assessment indicated that the Emergency Operations Center (EOC) had yet to assume a fully functional coordination role during outbreaks. Terms of reference and standard operating procedures remained incomplete, limiting its operational capacity. Incident Command System meetings occurred but lacked sufficient multidisciplinary engagement, preventing the EOC from fully serving as a central command hub.

Significant variation was observed in local government unit (LGU) engagement in cholera and WASH-related activities and in outbreak response mechanisms. In the Philippines, while DOH can recommend outbreak declarations at the LGU level based on epidemiological criteria, declaration authority rests with the local chief executives. This can cause the two levels to have different counts of epidemiological and declared outbreaks.

Progress in strengthening partnerships was noted through recent collaboration between the Department of the Interior and Local Government, the Philippine Information Agency and the Department of Social Welfare and Development. Informants highlighted the fact that the 2022 cholera outbreak in Samar had demonstrated improved collaboration, as local officials worked closely with health workers, and multisectoral teams were convened for joint investigations, comprising health, surveillance, laboratory, sanitation inspectors, and Food and Waterborne Diseases Prevention and Control Program staff.

Governance challenges hindered the implementation of WASH initiatives. Although Local Drinking-water Quality Monitoring Committees are mandated, many remained inactive or lacked clearly defined roles. WASH staffing shortages persisted, with sanitation inspectors overseeing populations above the recommended ratio of 1:20 000, (*17*) leading to heavy workloads and high turnover. A good practice identified in Samar involved village sanitation inspectors receiving modest honoraria from municipal budgets. Additionally, LGUs had not fully used available funding streams, such as for disaster risk reduction and management, to strengthen WASH programmes.

### Epidemiology and surveillance

Weekly case-based reporting to the Philippine Integrated Disease Surveillance and Response system was conducted in all assessment sites. Connectivity challenges at the LGU level limited use of the online reporting system, requiring double data entry and increasing the workload at the provincial and regional levels. In March 2024, the cholera case definition in Eastern Visayas was reverted to require signs of severe dehydration. Following this, surveillance staff observed a decline in health-care workers considering cholera in differential diagnoses. Event-based surveillance training was in progress during the risk assessment.

Confirmation of suspected cases remained limited across Eastern Visayas; awareness of Department Memorandum 2020–0391 on cholera RDTs was minimal, and RDT supplies at the RESU in the City of Tacloban were limited and released only upon request. Additionally, staff training on RDT use was ad hoc and mainly conducted during outbreaks, contributing to low awareness of the testing algorithm.

Specimen collection and transport protocols were established; however, procurement and logistical challenges constrained these activities. While the City of Tacloban had cholera confirmatory testing capacity, Cary Blair medium was unavailable, transportation was irregular, particularly in geographically isolated and disadvantaged areas, and field workers were unaware that the reagent was available from the City of Tacloban and reported sending samples to Manila for testing.

Municipality data management, analysis and reporting capacity were limited, with most analyses conducted at provincial or regional levels, contributing to delays. The RESU provided automated analysis and reports for priority diseases but lacked cholera-specific templates. Surveillance staff expressed a need for training to enhance their data analysis and interpretation skills, along with IT equipment to support surveillance operations. Few staff knew the criteria for officially closing an outbreak or conducting a risk assessment, which warrants training in these areas.

### Water, sanitation and hygiene

In most areas visited, water refilling stations were the primary drinking-water source, with families typically paying 25–30 Philippine pesos for a 20-litre container, despite daily incomes as low as 200 pesos. Those unable to afford water from refilling stations relied on a low-cost alternative, such as open wells, rivers, streams or unregulated vendors, and automated *tubig* (water) machines.

Poor water quality was evident in the sampling: all Level I and II samples (13/13) were positive for *E. coli* and total coliform, while Level III samples showed no *E. coli* (0/6) but 33% (2/6) were positive for total coliform (**Table 2**). Potential sources of contamination included proximity to septic tanks (< 25 m, uncontained solid waste, poor drainage and open defecation, particularly in coastal areas with limited toilet access.

**Table 2:** Water sampling results, by water source level, Eastern Visayas, Philippines, 2024

Local government unit	Water source	No. (%) of samples positive for *E. coli*	No. (%) of samples positive for total coliform
**Eastern Samar**	**Level I**	**8/8 (100)**	**8/8 (100)**
	**Level II**	**–**	**–**
	**Level III**	**0/4 (0)**	**2/4 (50)**
**Subtotal**		**8/12 (67)**	**10/12 (83)**
**Samar**	**Level I**	**3/3 (100)**	**3/3 (100)**
	**Level II**	**1/1 (100)**	**1/1 (100)**
	**Level III**	**–**	**–**
**Subtotal**		**4/4 (100)**	**4/4 (100)**
**City of Tacloban**	**Level I**	**1/1 (100)**	**1/1 (100)**
	**Level II**	**–**	**–**
	**Level III**	**0/2 (0)**	**0/2 (0)**
**Subtotal**		**1/3 (33)**	**1/3 (33)**
**All LGUs**	**Level I**	**12/12**	**12/12**
	**Level II**	**1/1**	**1/1**
	**Level III**	**0/6**	**2/6**
**Grand total**		**13/19 (68)**	**15/19 (79)**

LGU: local government unit.

Level I (point source) refers to a protected well or developed spring without a distribution system, serving around 15 households; Level II (communal faucet system) refers to a system with a piping network and communal faucets serving 40–100 households; Level III (individual house connections) refers to a system with household taps suitable for densely populated areas.

Water refilling stations and water districts met DOH requirements for an approved water safety plan under Administrative Order No. 2014–0027. However, other water supply sources managed by the LGU or community lacked established water safety plans.

The region had four DOH-accredited laboratories, which were insufficient for routine monitoring under the 2017 National Standards for Drinking-water. (*11*) Although portable water testing kits were available, resources for consumables, such as reagents, were often not earmarked by LGUs, contributing to delays and reduced accuracy. Most rural sanitation inspectors reported that they were trained to conduct essential water testing and analysis.

Qualitative insights from discussions with community members indicated that many septic tanks lacked bottom slabs, posing groundwater contamination risks. Official sanitation data often misrepresented conditions due to inconsistent interpretation of indicators, especially those defining safely managed sanitation services.

Open defecation persisted in some areas. For households with toilets connected to septic tanks, desludging costs (3500–6000 pesos), unregulated services and improper septic tank management created additional risks. Informal coastal settlements faced additional WASH-related challenges due to development restrictions. Hand hygiene remained limited by inadequate facilities and cost barriers, with informants reporting that while they understood its importance, they could not afford to keep soap available at handwashing stations specifically for that purpose.

### Risk communication and community engagement

Community members demonstrated a basic understanding of cholera symptoms and prevention, often using local terms such as *urosuka* (vomiting and diarrhoea) and *eltur* (cholera). They recognized the link between cholera and contaminated water and food and unsanitary conditions, and were aware of preventive measures, including drinking clean water, boiling water when necessary, covering food, using personal toilets, maintaining cleanliness and practising hand hygiene.

Community engagement activities aimed at enhancing cholera prevention knowledge and practices remained limited in both reach and consistency. Health workers served as the primary facilitators for RCCE initiatives, raising awareness through family development sessions under the 4Ps programme (which supports low-income households), village assemblies and community clean-up drives. The frequency and scope of these activities varied widely across LGUs, and mechanisms for community feedback remained minimal.

EVCHD developed key cholera messaging based on experiences, primarily disseminated through social media channels. Respondents expressed a preference for information, education and communication materials in the local Waray language. However, regional language variations posed challenges for consistent messaging. Colourful printed materials were preferred for their clarity and visual appeal.

High perceptions of cholera risk, shaped by past outbreaks, coexisted with inconsistent health-seeking behaviours. While fear of cholera's severity and hospitalization costs motivated preventive practices, individuals reported seeking health care only when symptoms worsened or when children were affected.

Health workers, particularly village health workers, were identified as highly trusted figures and served as the primary source of health information and as front-line emergency responders in the communities. They actively engaged in case investigation, referrals, the distribution of sodium hypochlorite solution and oral rehydration solution, health education and case monitoring, and expressed the need for additional training, job aids and resources to support RCCE activities.

## Discussion

This joint risk assessment conducted in Eastern Visayas in 2024 identified that partial progress had been made across key technical areas since 2016. Several good practices were documented, including a well established surveillance system with high-frequency reporting (daily during outbreaks and weekly during routine operations) and the demonstrated capacity of sanitation inspectors in water sample collection and handling. Additionally, a high level of cholera risk perception was observed among community members. Despite this progress, most recommendations from the 2016 assessment remained only partially implemented, indicating the need for continued strengthening across technical areas (**Table 1**).

Epidemiological trends in **Fig. 1**, including the decline in incidence after 2022, largely reflect changes in surveillance sensitivity. During the 2022–2023 outbreaks, a broader definition of a suspected case and active RDT use enabled more sensitive detection. In early 2024, the region reverted to the standard, more restrictive EDCS definition that required the presence of severe dehydration, reducing overall case detection. These changes, combined with the overall reduction in outbreak magnitude, contributed to the observed decline in reported incidence.

This assessment further highlighted, through interviews and field observations, the systemic and socioeconomic determinants influencing cholera risk in the Philippines. Effective prevention and response require multisectoral coordination and leadership. The devolution of health services under the 1991 Local Government Code established a decentralized system, with LGUs managing service delivery and DOH providing policy direction and technical support. (*18*) While decentralization has enabled localized implementation, it has also introduced challenges in procurement, training, outbreak declaration, incident management and interdepartmental coordination. This assessment also emphasized the impact of socioeconomic vulnerability, (*19*, *20*) particularly among coastal populations with informal or unstable employment and limited access to WASH services. Although these communities demonstrated high awareness of cholera prevention measures, their ability to act on this knowledge was limited by resource constraints.

### Limitations

This assessment had several limitations. First, as a predominantly qualitative study, it did not allow for a systematic evaluation of outbreak response timeliness. Second, reporting bias may have influenced findings; for instance, some self-reported handwashing durations exceeded 10 minutes, which appears to represent an overestimation in the context of limited water availability. Triangulation methods were employed to mitigate this, which included interviews with multiple types of respondents and, when possible, the integration of quantitative data, such as surveillance reports and water quality testing. Third, changes over time to the case definition of suspected cholera, particularly the reversion to a more stringent definition requiring severe dehydration after March 2024, may have affected case detection and limited comparability across different periods. Fourth, the assessment was limited to four municipal sites and one city site in Eastern Visayas, which may restrict the generalizability of findings to other regions in the Philippines.

### Conclusions

This risk assessment demonstrated that cholera remains a persistent public health threat in Eastern Visayas, driven by multiple interrelated factors, including limited outbreak response capacity, fragmented governance and insufficient access to WASH services. Addressing these challenges requires a coordinated, multisectoral approach, leveraging existing structures such as the EOC as an integrated response hub and ensuring effective coordination across surveillance, WASH and RCCE.

To address systemic gaps, the development and implementation of national strategies for cholera control should be prioritized to clearly define roles, responsibilities and priority areas across national and local levels. These strategies should be embedded within the broader national Food and Waterborne Diseases Prevention and Control Program framework to ensure coherence and sustainability. Securing formal, active support from local authorities will be essential in ensuring ownership and sustainability. The strategies should be aligned with the Global Task Force on Cholera Control framework. (*21*) Furthermore, in line with these findings and water testing results, prioritizing Level I and II water systems for water safety planning, strengthening routine water quality monitoring, and sustaining investments in WASH infrastructure are essential steps for reducing the long-term burden of cholera and other foodborne and waterborne diseases in highly vulnerable areas of the Philippines. (*22*)
