# Supplementary material for: Risk assessment of cholera outbreaks in Eastern Visayas, Philippines, 2024
Source: Western Pac Surveill Response J. 2026 Jul 14;17(3):1–12. doi: 10.5365/wpsar.2026.17.3.1271 (PMC13575672; doi:10.5365/wpsar.2026.17.3.1271)
Supplement: Supplementary file 1 [file wpsar-17-1271-s001.pdf]

Supplementary Material 1. Risk Assessment Checklist for Cholera Outbreak in Eastern Visayas: Surveillance and Epidemiology

Date:

Completed by:

Surveillance & Epidemiology (General Information)

Data source: Document review (surveillance reports) and interview with the Food and Waterborne Diseases (FWBD) Prevention and Control Program coordinator, Regional Epidemiology and Surveillance Unit (RESU) and Provincial Epidemiology Surveillance Unit (PESU)

Objective: Understand the context in which cholera cases are occurring and the policies and guidelines in place for detection and response

- **Case definitions - Cholera and acute watery diarrhoea (AWD)**

Case definitions and alignment across national, regional, provincial and municipal levels with WHO guidelines

	AWD	Suspected cholera case	Confirmed cholera cases
Nation			
Region			
Province			
Municipality			

Are they in line with WHO guidelines? Yes / No

If no, give possible reasons:

WHO definitions

Acute watery diarrhoea: Acute watery diarrhoea is an illness characterized by three or more loose or watery (non-bloody) stools within a 24-hour period.

Suspected cholera case:

- In areas where a cholera outbreak has not yet been declared, any person aged 2 years or older presenting with acute watery diarrhoea and severe dehydration or dying from acute water diarrhoea
- In areas where a cholera outbreak has been declared, any person presenting with or dying from acute watery diarrhoea

Confirmed cholera case: A suspected case with *V. cholerae* O1 or O139 confirmed by culture or polymerase chain reaction (PCR)

- **Indicators**

Types of indicators used/available for routine surveillance at regional/provincial/local levels (select all that apply)
• Incidence rate<br>• Attack rate<br>• Positivity rate of confirmatory diagnostic tests (culture or PCR)<br>• Case fatality rate (CFR)<br>• Others:

- **Context-specific vulnerability indicators**

What are the characteristics of this municipality related to the components below? (select all that apply)
• Coastal areas<br>• Mountainous areas<br>• Geographically Isolated and Disadvantaged Areas (GIDA)<br>• Transportation hub *Area along major travel routes with transportation hubs, such as bus terminals<br>• Areas at high risk for extreme climate and weather conditions *El Niño, heavy rains, floods or droughts<br>• Others:
**Is this area adjacent to other provinces with cholera outbreaks? Yes / No**
**What are the major population gatherings in this area?**
• Religious gatherings, such as worship or missa, or Jumu'ah (Friday prayers)<br>• Local markets<br>• Local festivals, such as wedding ceremonies<br>• Funerals<br>• Occupational-related population movement and settlement (seasonal agricultural and fisheries work or settlements)<br>• Others:

- **Data-sharing/reporting system**

With whom are data shared/reported
**Which data-sharing/reporting method is used**
• Which organization or unit is responsible?<br>• Which data management platform is used? (e.g. Google Sheets)<br>• Which reporting form is used? How are the results from the laboratory sent to other agencies? (e.g. via email, event-based surveillance and response [ESR] reports)<br>• How often are the data reported?<br>• What are the data-sharing and reporting streams?<br>• Guidelines/standard operating procedures (SOPs)

- **Data analysis at provincial/regional levels**

**Are you familiar with the methods used to identify the cause of a cholera outbreak? Yes / No**
If yes, please explain further.
**Have you received training on how to investigate or analyze the causes of a cholera outbreak? Yes / No**
If yes, please explain further.
**Which data analysis method is used?**
• Is data analysis performed on a regular basis?<br>• Which organization or unit is responsible?<br>• Which software program/methods are used for cleaning, mapping, tabulating and graphing?<br>• How often is the analysis disseminated?<br>• What is the data analysis process?<br>• Guidelines/SOPs

- **Surveillance at provincial/regional levels**

**Which types of surveillance are currently used for cholera? (select all that apply)**
• Health-care facility-based surveillance<br>• Genomic surveillance<br>• Community-based surveillance<br>• Event-based surveillance<br>• Surveillance in the Field Health Service Information System<br>• Surveillance in the Philippine Integrated Disease Surveillance and Response (PIDSR) system<br>• Laboratory-based surveillance<br>• Others:
**Health facility-based surveillance**
• Is all barangay data included in health facility surveillance?<br>• Is all health facility or hospital data included in PIDSR surveillance?<br>• Who is conducting and to whom are they reporting?<br>• How often is it conducted/reported?<br>• Are guidelines/SOPs for handover developed?

Event-based surveillance
• Who is conducting and to whom are they reporting?<br>• What is the percentage of total events created/reported for ESR reports?<br>• What is the ESR report reporting frequency in routine and outbreak situations: daily, weekly, monthly, quarterly or annually?<br>• Are guidelines/SOPs for handover developed?
**Community-based surveillance**
• Who is conducting and to whom are they reporting?<br>• How often is it conducted/reported?<br>• Are guidelines/SOPs for handover developed?
**FHSIS surveillance**
• Is all barangay data included in FHSIS surveillance?<br>• Who is conducting and to whom are they reporting?<br>• How often is it conducted/reported?<br>• Are guidelines/SOPs for handover developed?
**PIDSR surveillance**
• Is all health-care facility data included in PIDSR surveillance?<br>• Who is conducting and to whom are they reporting?<br>• How often is it conducted/reported?<br>• Are guidelines/SOPs for handover developed?
**Areas for improvement**
• Are there any identified areas for improvement in the surveillance system for cholera?

- **Surveillance staff training and capacity**

**Number of surveillance officers**
• How many surveillance staff are currently employed in this area? (includes permanent and job order staff with any role in surveillance and epidemiology)
**Training programmes**
• How many surveillance staff have participated in training programmes, such as the Field Epidemiology Training Program (FETP)? What level of training have they completed? How many people with FETP-adv are still involved in "on the ground" activities?
**Response to suspected cholera/AWD cases**
• Do the surveillance staff know how to respond when there is a case of suspected cholera or AWD?
**Use of cholera rapid diagnostic tests (RDTs)**
• Are the surveillance staff trained to use cholera RDTs? Yes / No
**Areas for improvement**
• Are there any identified areas for improvement in the training and capacity of the surveillance staff? Is there a plan already in place to address these training gaps?

- **Risk assessment**

**Risk assessment knowledge and experience**
• Has a risk assessment for cholera ever been conducted? Yes / No If yes, please describe.
**Criteria and guidelines**
• Are there any guidelines/SOPs for conducting a risk assessment? Yes / No<br>• If you have conducted a risk assessment, what criteria did you use? (e.g. probable cholera outbreak threshold or CFR)

- **Response measures**

**Are you aware of the Department of Health (DOH) guidelines? Yes / No**
• If yes, are your response measures in line with DOH guidelines?
**Field investigations**
• Proportion of cholera signals investigated within 24–48 hours at provincial/regional levels
**Case management in the community**
• If suspected cases are detected in the community, how are they managed?<br>• Who will manage them?<br>• Which process do they follow?<br>• Are there any guidelines/SOPs for this?

- **Case management in the health facilities**

**Patient flow chart**
**Admission and triage standards**
**Treatment SOPs**

Documents to request:

- Sit reps
- Other reports
- Line lists
- Spot maps (inc. photos at all health centres)
- FWBD action plans

Surveillance & Epidemiology (Response)
***Fill out for each cholera event separately, up to a maximum of three in one barangay**

Data source: Document review (field investigation reports) and interview with Food and Waterborne Diseases (FWBD) Prevention and Control coordinator, Regional Epidemiology and Surveillance Unit (RESU), Provincial Epidemiology Surveillance Unit (PESU), Municipality Epidemiology Surveillance Unit (MESU) and City Epidemiology Surveillance Unit (CESU)

Objective: Understand how cholera outbreak responses are conducted step by step in each outbreak, including key dates of actions, detection, testing, field investigation, reporting, risk assessment and outbreak declaration

- Name of province:
- Name of municipality:
- Name of barangay:

Key Dates

- First suspected case developed symptoms
- First suspected case reported to ESU (Notification)
- First RDT conducted
- First sample sent for lab testing
- First lab test result received from the lab
- First ESR shared
- Investigation team created
- Field visit conducted
- First field report shared
- Outbreak declared
- Outbreak closed

Early detection of cases

- From which data sources were suspected cholera cases first reported to health authorities? (select all that apply)

Health facilities	Laboratories	Community health workers
Teachers	Religious leaders	Local leaders
Media	Rumours	
Others:		

- How is the data source verified to determine whether reports of cases or outbreaks are accurate when suspected cholera cases are first reported? (e.g. immediate field investigation conducted to confirm the cases or outbreak)

- Was this area previously unaffected when suspected cholera cases were detected? Yes / No

- Before the expected season arrived, was surveillance initiated or strengthened in at-risk areas with known seasonal upsurges and recurrent outbreaks to detect the first cholera cases as early as possible? Yes / No

Definitions

- Have you applied same definitions from national/regional DOH regarding AWD, suspected/confirmed cholera cases at the barangay? Yes / No

- If no, please explain the definitions used here

AWD	Suspected cholera case	Confirmed cholera cases

Use of cholera rapid diagnostic tests (RDTs)

- Do you use RDTs for screening to facilitate early outbreak detection? Yes / No

- Have RDTs been used to triage specimens for laboratory testing or in place of stool culture or polymerase chain reaction (PCR) to confirm cholera before? Yes / No

- Was a cholera outbreak only confirmed when specimens from suspected cases tested positive by culture or PCR at the reference laboratory? Yes / No

- Do you use cholera RDTs for other purposes besides outbreak screening (e.g. routine testing, research)? Yes / No

  If yes, please indicate for which other purposes cholera RDTs are used.

Investigation of the alert, risk, needs assessments and initial response

- Was an investigation team deployed quickly, preferably within 24 hours, to address the situation? Yes / No If no, were there any barriers that prevented it?

- Was an investigation team dispatched to the field to investigate every cholera alert at the barangay level to confirm or rule out the outbreak? Yes / No

- If no, were there any barriers that prevented investigations for every cholera alert?

- What proportion of investigations were conducted out of all cholera alerts at barangay level?

- What was the team composition? (select all that apply)

Doctor experienced in cholera case management	Epidemiologist	Water and sanitation expert
Infection prevention and control expert	Expert in social mobilization	Community engagement and risk communication
Laboratory technician for stool collection from suspected cases	FWBD programme staff	Barangay Health Worker
Health education and promotion officer	Others:	

- What were conducted by the investigation team? (select all that apply)

Assess the risk of spread	Identify priority actions	Conduct an initial needs assessment
Implement initial control measures	Collect samples for testing	Conduct RDT for suspected cholera cases
Investigate transmission mode/ sources	Create a line list of the suspected cases	Interview suspected cases
Others:		

- How many days did it take to report findings, including risks and assessed needs, to decision-makers to provide a rapid and focused response? Were any challenges/delays experienced?

- What supplies did the team carry to the field? (select all that apply)

Human specimen collection and transport supplies	Antibiotics	Oral rehydration salts (ORS)
Water testing supplies	Protocols	Other water, sanitation and hygiene (WASH) investigation supplies
Others:		

- What was the transmission mode/sources investigated of cholera suspected cases/outbreak?

Contaminated drinking water (water source)	Contaminated food	Contaminated environment (e.g. pigsty)
Poor sanitation/hygiene practices	Direct contact with other suspected cholera cases/ outbreaks	Unknown
Others:		

Risk assessment

- Was a risk assessment conducted to assess the risk of further transmission (spread, magnitude, capacity to control outbreak) and potential impact of the outbreak? (e.g. a comprehensive or rapid risk assessment beyond the ESR risk assessment questions) Yes / No

  If yes, then:

- What was the likelihood of transmission?
  (e.g. based on factors such as access to safe water and improved sanitation; population behaviour [including water sources used, chlorination, open defecation, handwashing, barriers to protective behaviours]; geographical, environmental and climate conditions [expected cholera season, expected weather patterns, flooding, drought]; areas with high population density; and areas with high transit of people or an influx of travellers)

- What was the potential impact of the disease?
  (e.g. based on factors including existing cholera preparedness, access to treatment such as ORS and intravenous [IV] fluids, capacity of health-care workers to provide case management, available supplies, health-seeking behavior, malnutrition status, and population immunity, as determined by previous exposure to cholera or previous cholera vaccination)

Needs assessment

- Has a needs assessment been conducted to identify the available resources (human and supplies) and estimate the needs based on the risk assessment? Yes / No

  If yes, then:

  - Were these estimates communicated quickly to local and national authorities so the necessary resources could be rapidly procured and/or provided by the government or partners? Yes / No
  - Were the supplies needed calculated based on the expected attack rates (ARs) and the population? Yes / No
  - Were the above actions taken before an outbreak was confirmed or declared? Yes / No

- **Outbreak confirmation**

Definitions

- What is the definition of cholera outbreak?
- Was a cholera outbreak declared for this cholera event? Yes / No
  Why or why not?

Admission and triage standards

- When a cholera case was suspected and an alert was triggered, were stool samples collected from symptomatic individuals and sent to the reference laboratory for microbiological confirmation by culture and/or PCR? Yes / No
- If yes, was antimicrobial susceptibility testing conducted?
- For each new barangay suspected to have a cholera outbreak, was laboratory confirmation of specimens from suspected cholera cases conducted by microbiological culture or PCR? Yes / No
- How many stool specimens were collected from the first suspected cases (5–10 cases) and sent to the laboratory for confirmation?
- Once a cholera case was detected, were all suspected cases sampled for lab confirmation? Was the clinical case definition alone sufficient to monitor epidemiologic trends?

Collection, storage and transport of samples for confirmation by culture and PCR

	Number	Notes
Total suspected cases		
Cases with faecal specimen collected within 4 days of illness		
Cases with specimen collected before antimicrobial therapy begun		
Cases with both specimen collection within 4 days and before antibiotic treatment		

- Which kind of transport medium was used to collect and preserve the stool samples?
- Average duration between
  - Sample collection and sending it to the lab (hours/days)
  - Receipt at the lab
  - Receipt of results

Reporting of laboratory results

- Did laboratories maintain an updated database with all samples received and tested, and the results of testing?
  Yes / No

- Did laboratories send alert results to attending clinicians upon completion of initial testing steps and suspicion of cholera (at laboratory level) while waiting for culture results? Yes / No

- Did laboratories send results immediately after the completion of confirmatory testing to related health authorities? Yes / No

- **Organization of the response**

Activate the cholera coordination committee

- Was a multidisciplinary cholera coordination committee created for coordination between relevant sectors?
  Yes / No

  If yes, please list all the areas of expertise/programme areas that were included in the cholera coordination committee (e.g. WASH, education, risk communication and community engagement, hygiene promotion) with representatives from relevant departments and local authorities, international agencies, nongovernmental organizations and others, as appropriate?

Develop a cholera response plan and a preparedness plan for areas at risk

- Was an integrated and multisectoral cholera response plan developed based on risk and needs assessments?
  Yes / No

  If yes, please explain further.

Implement control measures

- What control measures were implemented?

setting up cholera treatment facilities	distributing ORS	employing early detection and triage	informing the public
training health professionals	distributing treatment protocols	estimating and procuring supplies	

- How quickly and for how long were these control measures implemented? Was there a specific duration criterion for sustaining these measures?

Cholera kits

- Were any cholera kits used? (e.g. treatment kits, provisional structure kits, outbreak investigation kits, laboratory confirmation kits)

- How was the procurement of the cholera kits carried out?

- Is there a testing strategy in place for the use of these kits?

- **Monitoring the outbreak**

Epidemiological surveillance

- How was epidemiological monitoring for the outbreak being carried out?
- What types of data were being utilized, and what analyses were being conducted? (e.g. description of the outbreak; conducting field investigations including active case finding; exploring possible sources of contamination, and identifying risk factors and transmission pathways; testing sources of drinking water for fecal contamination or free residual chlorine (FRC); mapping locations of homes and water sources of cholera patients)

Data collection

- Was a standard line list available and used in all health facilities, with the same data being collected by all partners?
- Was a template provided if necessary?
- Were the registers paper-based or electronic-based forms?
- Which case information was included?

name	date of visit	outcome	stool sample taken
age	dehydration status	date of discharge or death	lab confirmation (culture or PCR)
sex	treatment plan		vaccination status
home address	hospitalization	RDT result	exposures
pregnancy	malnutrition		occupation

- Was data compiled daily and reported to the local health authorities through established channels? Are patient records and registers kept from the start of the epidemic to its very end?
- Was community-based surveillance implemented in areas where necessary? Yes / No
  If yes, please explain further.

Data reporting/analysis

- Did you have data validation processes? Yes / No
  If yes, please explain further.
- Which data was reported to the surveillance unit/health authorities' office at provincial, regional, or national levels? (e.g. the aggregated number of cholera cases and deaths — both registered at the health facility and occurring in the community — by age group, minimally under 5 years of age and 5 years of age or older, etc.)
- What analysis is conducted to determine the likely source of the cholera?
- What was found to be the likely cause of this outbreak/cholera case?
- Were response measures implemented in response to the identified source of cholera?

Closing the event

- How was the cholera outbreak/event closed?
- Is there a set criteria for closing the outbreak? Was there a requirement for testing?

Follow-up

- Since the closing of the cholera outbreak/event in this area, have there been changes to:
    - Routine surveillance
    - Preparedness for future suspect cases of cholera
    - Additional trainings to support early detection of cholera
    - Others:

Laboratory

Data source: Document review (laboratory-testing guidelines) and interviews with laboratory personnel, the laboratory focal point from the Regional Epidemiology and Surveillance Unit (RESU) and the Provincial Epidemiology and Surveillance Unit (PESU)

Objective: Understand the laboratory testing system where cholera cases are identified, and the policies and guidelines in place for testing and confirmation

Some questions appear to be duplicated in the epidemiology/surveillance section; however, please focus on the system, data sharing, and reporting regarding laboratory information.

- **Laboratory confirmation process for cholera**

**Describe the process/flow for confirming a suspected cholera case**
• The method of collecting stool samples from symptomatic individuals<br>• Minimum number of specimens required for PCR<br>• Minimum number of specimens required for culture<br>• Storage of samples<br>• Transport of sample process<br>• Types of testing, such as culture and/or PCR and antimicrobial susceptibility testing
**Are there any guidelines/SOPs for the confirmation process? Yes / No**
If yes, please list them:
**What kinds of testing/microorganism are done for suspected cholera cases?**
**What is the average turnaround time for cholera testing from the moment the specimen is received at the laboratory to the release of results?**
**Are there enough staff to cover all required testing? Yes / No**
If no, what’s the barrier to securing enough staff?

**Are there training programmes for capacity-building of staff? Yes / No**
If yes, please explain further.
**Do they provide education/training for RDT to related health offices/workers? Yes / No**
If yes, please explain further.

- **Data sharing**

**Do laboratories routinely report to local health authorities? Yes / No**
**Is the antimicrobial susceptibility profile reported to guide cases management? Yes / No**
If yes, please explain further.
**Which data-sharing method is used?**
• Which organization or unit is responsible?<br>• Which data management platform is used? (e.g. Google Sheets)<br>• Which reporting form is used? How are the results from the laboratory sent to other agencies? (e.g. via email)<br>• How often are the data sent?<br>• What are the data-sharing and reporting streams?<br>• Guidelines/SOPs

- **Data analysis**

**Are case-based epidemiological data and microbiological data merged? Yes / No**
**Which data-sharing method is used?**
• Is data analysis performed on a regular basis?<br>• Which organization or unit is responsible?<br>• Which data analysis software is used? (e.g. RStudio)<br>• How often is the analysis disseminated?<br>• What is the data analysis process?<br>• Guidelines/SOPs

Supplementary Material 2. **Risk Assessment Checklist for Cholera Outbreak in Eastern Visayas: Water, Sanitation and Hygiene**

Date:

Completed by:

Water, Sanitation and Hygiene

- **Source of water supply**

**What is the main drinking water source for drinking water in your community?**
• Spring (Undeveloped, No cover)<br>• Spring (Developed, Covered)<br>• Open dug well<br>• Handpump<br>• Tap (from Water district or LGU-managed or community-managed water systems)<br>• Water refilling station
Are you informed by the Sanitary Inspector if such sources are safe to drink? Yes / No
**What do you do if there is no information on the quality of the water?**
• Boiling ______________How many minutes? .... • Use chlorine tablets<br>• Use hyposol _____________ What % concentration? 1.25% 3%<br>• Nothing<br>• Others, please specify:
**What is the major water source for household use in your community, such as for bathing, laundry and washing dishes?**
• Spring (Undeveloped)<br>• Spring (Developed)<br>• Open dug well<br>• Handpump<br>• Tap (from Water district or LGU-managed or community-managed water systems)
**Are there potential sources of contamination within 25 metres of the water sources (per DOH guidelines for safe distance from Code on Sanitation)? Yes / No**
If yes, what are those?<br>• Poultry<br>• Piggery<br>• Toilet/septic tank<br>• Open dump sites<br>• Landfill<br>• Open defecation<br>• Others:

- **Open defecation**

**Is there open defecation in this area? Yes / No**
**How many households do you think have no toilets?**
**Is there a zero open defecation programme being implemented in this area? Yes / No**

- **Water supply and sanitation facilities**

**Do water supply facilities used by the community have regular (at least once a year) water quality testing? Yes / No**
If no, give possible reasons:
**Describe accessibility of the household to water supply facilities**
• Within the household premises: Yes / No<br>• Water service availability is 12 hours or more per day: Yes / No<br>• Water has no *E. coli*: Yes / No
**Describe the accessibility of the household to sanitation facilities**
• Toilets are shared with other family/household: Yes / No<br>• Toilets are owned by individual family/household: Yes / No<br>• Toilets have septic tanks: Yes / No If yes, sludge from septic tanks are desludged and safely treated and disposed: Yes / No

- **LDWQMC (Local Drinking Water Quality Monitoring Committee)**

**Is the LDWQMC already organized? Yes / No**
If yes,<br>• In what year it was organized? __________<br>• Is there an executive order or any LGU policy issuance for LDWQMC? Yes / No<br>• What are the current activities of the LDWQMC?
**Is there a LDWQSP (Local Drinking Water Quality Surveillance Program) at the municipality? Yes / No**
**How many barangays are covered by LDWQSP?**
**What LDWQSP activities are being conducted?**
• Sanitary survey<br>• Risk assessment<br>• Water quality testing, interpretation and analysis<br>• Water quality interventions<br>• Others:

**Are guidelines/SOPs for LDWQSP available? Yes / No**
If yes, please explain further.
**Is there enough LGU funding allocation/support to sustain LDWQMC and LDWQSP activities? Yes / No**
If yes, please explain further.
**Is there a high turnover of personnel involved in WASH?**
• Estimated % of turnover per year: ______ %<br>• Reasons for turnover:

- **Water Safety Planning**

**Does the water supply provider have a DOH-approved water safety plan? Yes / No**
If yes,<br>• Year of approval _________<br>• Is the water safety plan being monitored? Yes / No<br>If no, give the reasons:

- **Food Safety**

**Is there a food safety programme at the community level? Yes / No**
**What food safety practices are applied in the community?**
• Handwashing: Which water supply?<br>• Separating raw food from cooked food<br>• Observing correct temperature for cooked food<br>• Safe storage for cooked food<br>• Cleaning and sanitizing of cooking utensils<br>• Others:
**How is adherence to the food safety programme monitored?**
• Regular inspections<br>• Community reporting mechanisms<br>• Others:

- **Community education**

**Are there community education programmes on household water treatment and safe storage methods? Yes / No**
If yes, please explain further.
**Are there community education programmes on food safety? Yes / No**
If yes, please explain further.

- **WASH and infection prevention and control (IPC) measures at the health facility**

Availability of protocols/guidelines for WASH and IPC at the health facility
**Availability of WASH/IPC supplies**
• Buckets<br>• Mops<br>• Detergents<br>• Hand soaps<br>• Alcohol-based hand rubs<br>• Chlorine or other disinfectants<br>• Brushes<br>• Personal protective equipment (PPE)<br>• Others:
**What is the process for ensuring the availability of supplies?**
• Annual budget allocation from the municipal government<br>• Inventory management<br>• Donations
**Are there any training programmes for staff on WASH and IPC measures? Yes / No**
If yes, please explain further
**How is staff compliance with WASH and IPC measures monitored?**
• Regular inspections<br>• Feedback mechanisms
**Is there a health-care waste management system in the facility? Yes / No**
If yes, please explain further
**Are there any challenges faced in implementing WASH and IPC measures?**

- **Management/treatment of water quality**

How often are water quality tests conducted at the health-care facility?
• Frequency of free residual chlorine (FRC) testing<br>• Frequency of fecal contamination testing
**What mandatory parameters are being tested?**

What are the protocols for responding to water quality test failures?
**What are main methods for household water treatment?**
• Thermal treatment (e.g. boiling)<br>• Chemical disinfection (e.g. chlorination)<br>• Others:
**Are water laboratory facilities available in the area? Yes / No**
If yes, please explain further.
**Are portable water test kits available in the area? Yes / No**
If yes, please explain further.

Supplementary Material 3. **Risk Assessment Checklist for Cholera Outbreak in Eastern Visayas: Risk Communication and Community Engagement**

Date:

Completed by:

Risk Communication and Community Engagement

- **Health literacy/education in the community (rapid assessment of community knowledge, attitudes, perceptions, behavioural barriers including structural, drivers, levels of trust and social norms that could impact cholera transmission)**

**How much knowledge of cholera does the community have?**
• Characteristics of cholera<br>• Mode of transmission<br>• Prevention and control measures
**What are the attitudes and perceptions of the community towards cholera and WASH practices?**
(probe about risk perception)
**What are the current behaviours and practices of the community when it comes to WASH?**
**What is the level of trust of the community towards the government? Health workers?**
• Towards the government<br>• Towards health workers
**What are social norms, cultural, and structural barriers in the community that could possibly impact cholera transmission?**
(probe about other barriers, e.g. not having information/knowledge, access, motivation)
**Do related health offices/workers have the data about the level of knowledge of cholera in each affected community? Yes / No**
• If they have data, how often do they conduct data collection and which methods are used?<br>• Which health offices/workers are in charge of it?

Do related health offices/workers have community information and education programmes on cholera for households? Yes / No
• If yes, are the programmes different depending on each community's level of knowledge or context?<br>• Who teaches the programmes?<br>• How often are the programmes conducted?<br>• What channels do they use to deliver the programmes?

- **Community health workers**

Main tasks of community health workers for cholera response
**Guidelines/SOPs/training for community health workers for cholera response**
**Level of capacity of health promotion workers on risk communication, community engagement and infodemic management**

- **Risk communication materials (map existing risk communication, community engagement and infodemic management (RCCE-IM) materials and identify gaps)**

Have messages for risk communication been developed according to the specific characteristics of the affected community? Yes / No
If yes,<br>• Which process have they used to develop the messages? Were these messages tested with communities?<br>○ Are there any references/guidelines for the development of key messages? Yes / No<br>• Have the messages been adapted depending on the local context and cultural nuances? Yes / No<br>• Are the messages composed of limited text, and contain illustrations of practical demonstrations and aligned with ongoing cholera prevention programmes? Yes / No

Have health and hygiene promotions or information, education and communication (IEC) materials been conducted previously to engage with the community? Yes / No
If yes, please explain further.
**What types of communication channels or dissemination methods are used?**
• Radio<br>• TV<br>• Press releases<br>• Social media<br>• SMS<br>• Posters<br>• Flyers<br>• Interpersonal communication, such as barangay leaders or religious leaders<br>• Talks in places where people usually gather, such as health-care facilities, churches, markets
**Are there feedback channels in place that can be used by at-risk communities? Yes / No**
• If yes, please explain further.<br>• If no, what's the barrier to using them?
**Communication channels/frequency at regional/provincial/local levels**

- **Infodemic management**

What are some of the misguided health practices in the affected community?
**Is there any fake news spreading regarding cholera? Yes / No**
If yes, please explain further.
**Is there any delivery ORS, soap for handwashing and products for water treatment in the community? Yes / No**
• If yes, is this being correctly used in the community?
**What are the common questions and concerns of the community regarding cholera?**

- **Civil society organizations (CSOs)**

What are the relevant and trusted CSOs that can support community-based activities in this area?

Collaboration with other agencies/partners

Data source: Interviews with CHD-8

Objective: Understand the status of collaboration with other agencies and partners

- **Collaboration with other agencies/partners**

Which governmental sectors are collaborating currently?
**Which international agencies/partners are collaborating currently?**
**Which local agencies/partners are collaborating currently?**

- **Development of Cholera Response Plan**

Are there cholera/FWBD action plans at regional/provincial/local levels? Yes / No
If yes, please explain further.
**Are these plans aligned with the national cholera plan or national FWBD plan? Yes / No**
If no, what's the barrier to aligning with the national cholera plan or national FWBD plan?
**Which local agencies/partners are collaborating currently?**

- **Coordination**

Who are the decision-makers for cholera activities?
**Who is coordinating the cholera plan or activities?**
**Do they have their own indicators to monitor and evaluate the cholera activities? Yes / No**
If yes, please explain further.
**What are the challenges faced in coordinating with other agencies/partners?**
